# The impact of aging on interhemispheric transfer time and respective sex differences

**DOI:** 10.1016/j.nbas.2022.100040

**Published:** 2022-04-04

**Authors:** David Riedel, Tim Fellerhoff, Andreas Mierau, Heiko Strüder, Dominik Wolf, Florian Fischer, Andreas Fellgiebel, Oliver Tüscher, Bianca Kollmann, Kristel Knaepen

**Affiliations:** a Institute of Movement and Neurosciences, German Sport University Cologne, Am Sportpark Müngersdorf 6, 50933 Cologne, Germany; b Department of Exercise and Sport Science, LUNEX International University of Health, Exercise and Sports, 50, avenue du Parc des Sports L-4671 Differdange, Luxembourg; c Department of Psychiatry and Psychotherapy, University Medical Center Mainz, Untere Zahlbacher Str. 8, 55131 Mainz, Germany; d Center for Mental Health in Old Age, Landeskrankenhaus (AöR), Hartmühlenweg 2-4, 55122 Mainz, Germany; e Leibniz Institute for Resilience Research, Wallstraße 7, 55122 Mainz, Germany

**Keywords:** Interhemispheric transfer time, Corpus callosum, Event-related potentials, Aging, Sex

## Abstract

Age-related cognitive decline has been attributed to degeneration of the corpus callosum (CC), which allows for interhemispheric integration and information processing [22,69]. Along with decreased structural integrity, altered functional properties of the CC may cause impaired cognitive performance in older adults, yet this aspect of age-related decline remains insufficiently researched [59]. In this context, potential sex-related differences have been proposed [31,58]. A promising parameter, which has been suggested to estimate functional properties of the CC is the interhemispheric transfer time (IHTT), which is ideally obtained from event-related potentials (ERP) evoked by lateralized stimuli [45]. To examine the possible functional consequences of aging with regards to the CC, the present study investigated the IHTT of 107 older (67.69 ± 5.18y) as well as of 23 younger participants (25.09 ± 2.59y). IHTT was obtained using an established letter matching task and targeting early N170 ERP components at posterior electrode sites. The results revealed significantly elongated IHTT in older compared to younger participants, but no significant sex differences. Furthermore, there was a significant positive correlation between IHTT and age, predominantly driven by the female participants. The present findings add support to the notion, that IHTT is subject to age-related elongation reflecting impaired interhemispheric transmission. Age-related decline in women appears to occur at a different age range compared to men.

## 1 Introduction

Though prone to some degree of fluctuation, aging has been shown to correlate with cognitive decline in a general sense, such that most cognitive domains like memory, speed of information processing, attentional function or even perceptual speed have been shown to be affected in some way [55], [22], [74]. This age-related decline has been accepted as one of the major challenges to our modern, ever aging societies. The question whether this decrease in cognitive performance is attributable to a rather global impact on the central nervous system or may be grounded in a local structural disruption remains unanswered, just like the distinction between normal aging and pathology is difficult [22].

However, reduced functionality of the corpus callosum (CC) can be regarded a likely factor [55]. This structure, which is comprised of more than 200 million white matter fibers, is the main connection between the two hemispheres of the human brain [20]. Studies of disrupted or degenerated CC have displayed its vital role with regards to interhemispheric integration when faced with tasks, which require resources from specialized brain regions in opposite hemispheres [3], [20]. Thus, it is conceivable that impaired functioning of the CC may result in a reduction of integrability of sensory-motor as well as cognitive operations and ultimately widespread cognitive decline. A general structural degradation of the CC related to normal aging is widely accepted [49], [25] and the notion of altered functional recruitment of both hemispheres has been suggested in this context [65], [59]. Although the underlying mechanisms remain largely unknown, some evidence for decreased cognitive performance related to altered structure of the CC due to aging has been gathered. For example, two investigations found white matter integrity obtained by diffusion tensor imaging to correlate positively with executive functioning [47] and memory performance [51]. Even longitudinal data suggest a positive relationship between structural integrity of the CC and cognitive performance in the context of aging [66].

In general, there is substantial debate over potential sex-related differences with regards to morphological aspects of the CC. Some argue that differences are negligible, when accounting for overall differences in brain size between men and women [18], while others acknowledge sex-related discrepancies [56]. Nonetheless, sex-related differences of structural properties of the CC have been observed and may be of importance in the light of the present study. These concern morphological aspects, such as volume or cross-sectional area [64], [9] as well as microstructural differences with regards to structural integrity of the CC [71], [25], [42], [36], [34]. White matter integrity of the CC might be greater in women than men before age-related degradation commences [34]. However, accelerated or earlier onset of degeneration of the CC in women compared to men has been suggested [58], [72], [36], while others found no sex-related difference [25], [28]. Thus, a potential influence of sex on age-related alterations of the CC’s structure has to be taken into account.

Clearly, aging can have some negative impact on the CC’s structure, which likely leads to impaired cognitive performance, however, less research has been done targeting functional aspects in this context [59]. The CC has been identified to act as a communicator between the hemispheres, thus, modulating brain functioning of interhemispheric processing by inhibitory or excitatory mechanisms [20], [45], [69]. Consequently, the functional properties of the CC itself might play an important role in the context of age-related decline of cognitive abilities [59]. One important aspect of interhemispheric transmission is its speed and the estimation of interhemispheric transfer time has been subject to a number of investigations [40].

### Interhemispheric transfer time (IHTT) as a measure of CC functioning

The concept of IHTT as a functional measure for interhemispheric integration was first proposed early in the twentieth century [52]. The idea was to compare manual reaction times with the stimuli laterally presented to only one visual field, such that in one condition information would have to be transferred to the opposite hemisphere in order to respond (i.e., crossed), while in the other condition ipsilateral processing was sufficient (i.e., uncrossed). The difference between manual reaction times (i.e., crossed-uncrossed difference, CUD) was supposed to reflect IHTT and was found to be around 3 ms [45].

The initial methodology using manual reaction times has been criticized, because such low or even lower values are incompatible with more recent estimates of conduction velocities in callosal fibers [24], [63] and even paradoxical negative CUDs have been observed [59]. Furthermore, transfer does not only appear to occur for single or specific processes, but rather at different speeds on different levels [27], such that differences in reaction times may be a rather crude and vague measure of IHTT with other paths and mechanisms involved than transmission via the CC exclusively [26].

Using the latencies of event-related potentials (ERP) in electrophysiological investigations has shown more valid, reliable and plausible results, i.e. longer IHTT, than the behavioral approach [37], [43], [45]. Often these studies employ lateralized visual stimuli and define IHTT as the difference between the latencies of early ERP components at homologous electrode sites [73], thus avoiding some of the drawbacks of the CUD method [45].

Although the precise mechanisms of interhemispheric processing are not fully understood, the CC appears to play an essential role with regards to IHTT. For example, evidence from investigations on acallosal patients have shown, that the disruption of the CC leads to heavily elongated CUDs [11], [41]. Additionally, these patients show a lack of the ipsilateral ERP [8], which is usually observable in healthy participants. This further indicates the involvement of the CC with regards to IHTT, especially when based on ERPs [40]. Finally, there is evidence from fMRI studies indicating interhemispheric transmission to be a relevant mechanism in the context of visual processing [68]. Thus, the ERP method has been deemed an adequate measure directly reflecting functional properties of the CC, which appear to be related to the structural integrity of the CC [73].

### The impact of age and sex on IHTT

Not much research has been conducted on the effects of age and sex on IHTT as an indicator of CC functionality. Conceptually, IHTT has been expected to decline related to the aging process [65], [6]. Studies employing the CUD method have mostly demonstrated elongated IHTT in older subjects [31], [55], [3], [62], while a few found no influence of age [38], [61]. Sex differences with the age effect driven by the female participants have been found [31] but could not be confirmed for visuo-motor measures of IHTT [3].

Data relying on the ERP method are less conclusive. An early study found no difference in IHTT analyzing P100 and N160 components at the PO3 and PO4 electrodes between an older and a younger group [23]. Contrary to their own prediction of increased IHTT in older subjects, another study found faster IHTT for the P1, but not the N1 ERP component at P7 and P8 electrodes in older participants [6]. A third investigation revealed increased latency in the ipsilateral ERP component in the older group, which may be indicative of increased IHTT [13]. A more unconventional approach analyzing ERPs across posterior electrode clusters in constrained frequency bands on a single-trial basis revealed elongated IHTT in the theta band driven by shorter contralateral N1 latency and no difference in CUD measures in older participants, concluding that age does not appear to affect IHTT [59]. To the best of our knowledge, no study has included potential effects of sex in the context of aging into their ERP-based analyses. In younger subjects shorter IHTT estimated from parietal electrodes was found in female subjects compared to males [43], while others found no difference [44].

The few studies researching IHTT in the context of aging are methodologically insufficient as well as inconclusive. Furthermore, the likely impact of sex has thus far not been taken into account. Consequently, the present study aims to fill the gap in the literature investigating IHTT in the context of aging and sex-differences using an established ERP paradigm and analysis methods, while including a larger sample size than previous investigations. Group comparisons as well as within-group correlations analyzing IHTT, age and sex will add to our understanding of the mechanisms by which IHTT is modulated and potentially indicate its role in the context of cognitive aging.

## 2 Materials and methods

The data of the present investigation were collected and analyzed at the German Sport University Cologne (GSU) as one of the research centers of the AgeGain research project [75]. The current study is an extension of the AgeGain project and separately funded by GSU internal funding in order to answer further related research questions. Thus, to explore the relationship between IHTT, age and sex, additional electrophysiological investigations were conducted as well as a younger group of participants were included. The original AgeGain research project as well as the extended investigation were approved by respective ethics committees.

### Subjects

To receive an extensive insight into the effects of age and sex on IHTT, two groups of subjects were recruited. The first included 107 subjects (m: 64, f: 43) aged 67.69 ± 5.18 years drawn from the AgeGain study sample representing the older population. Additionally, a younger group of 23 subjects (m: 14, w: 9) at 25.09 ± 2.59 years of age was recruited. All subjects gave their written informed consent. A full description of the AgeGain study, including further information on inclusion and exclusion criteria has been published in the study protocol [75]. Exclusion criteria mainly consisted of history of cognitive, neurological, psychiatric or cardiovascular illnesses. Furthermore, with regards to this investigation all participants were right-handed requiring a minimum score of +60 at the German version of the Edinburgh Handedness Inventory [48] in order to reduce asymmetry effects and increase comparability [5], [40]. They also showed normal or corrected to normal eyesight verified using Landolt broken ring testing [57].

### Task

**Fig. 1:**
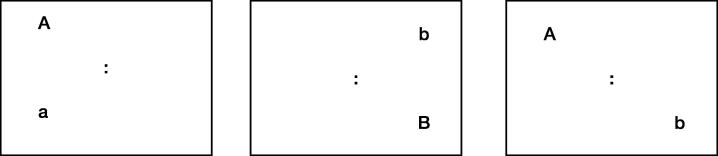
Schematic illustration of the task. Panel A provides an example for a matching trial with the stimuli presented to the left visual field. Panel B displays a matching trial with the stimuli in the right visual field accordingly. Panel C exemplifies a non-matching trial with the stimuli presented to both visual fields.

Applicable recommendations with regards to divided visual field paradigms were followed closely [5]. Participants were asked to perform an adapted Dimond paradigm, [17], [7], which has been used in a number of other studies [37], [43], [12]. Prior pilot testing yielded more unequivocal ERPs for the Dimond paradigm than for the Poffenberger paradigm, while also allowing for further exploratory analyses. Subjects were seated in front of a MacBookPro13,2 (Apple Inc., Cupertino, USA) with a screen resolution of 2560x1600 pixels and frame rate of 60 Hz. Their head was placed on a chin rest, such that eyes were kept level with the center of the screen at a distance of 57 cm. Normal room illumination with the windows sealed with blinds was used in order to avoid visual fatigue and conditions were kept the same between participants. The task was administered using PsychoPy 1.84.2 [50]. Instructions were given in written form and illustrations as well as verbally and 20 practice trials were performed. A fixation symbol in form of a colon was presented to the center of the screen and indicated the start of each trial by flashing once. Two of four letters (A, a, B, b), one lowercase and the other uppercase, were displayed on the screen in two of four positions around the center at random (Fig. 1). Lateral visual angle of the inside edge of the stimuli from the center was set to 3°, vertically to 1.5° and the height of the letters was about 0.5°. The stimuli appeared 1.50, 2.25 or 3.00 s after trial onset for an exposure duration of 150 ms. Any text displayed to the screen was black on a gray background. This had been found to be the least fatiguing during pilot testing. Subjects were asked to decide if the letters were the same (Aa/Bb) or different (Ab/Ba) and to respond accordingly with either the index (match) or middle finger (non-match) as quickly and accurately as possible. Responses were collected using a response pad (Cedrus RB-540, Cedrus Corporation, San Pedro, USA). This resulted in three conditions with both stimuli in the left visual field (LVF), both in the right visual field (RVF) or one stimulus in each visual field (BVF). The latter further encouraged central fixation. The task consisted of 14 blocks of 24 trials each with a 20 s break between blocks. It was performed once for each hand [12], which resulted in a total duration of 35–40 mins. The succession of hands used across the experiment as well as of the conditions of the trials within each block were random ensuring balance.

### Recording of event-related potentials

While performing the task, EEG was recorded continuously using a 64-electrode ANT system (asalab, ANT Neuro, Hengelo, Netherlands) with the electrodes arranged according to the international 10–10 system [33]. Two further electrodes (Blue Sensor N ECG Electrodes, Ambu, Bad Nauheim, Germany) were placed horizontally next to the lateral canthus of each eye for electro-oculography (HEOG) recording. Impedance of each electrode was kept below 20 kΩ [24]. Digital trigger signals indicating the trial’s condition were sent to the amplifier using a LabJack U3 (Labjack Corporation, Lakewood, USA). Additionally, a custom-made light sensor was used to accurately detect the stimulus onset. This was achieved by sending analog triggers set off by the refreshed frame toggling a rectangle in the bottom right corner of the screen, but covered by the sensor, from black to white at stimulus onset. ASA4 software (ANT Neuro, Hengelo, Netherlands) was used to record all electrophysiological and trigger signals at 1024 Hz, which were eventually exported to BrainVision Analyzer files (Brain Products GmbH, Gilching, Germany) for further analysis. Data was band-pass filtered to 0.5–30 Hz and a 50 Hz notch filter was applied. Bad channels were interpolated using spherical spline interpolation. Data was then stimulus locked and segmented to −300 ms – 700 ms and only trials with correct responses were kept for further analyses [37], [12]. After automatically rejecting any trials with artifacts (maximal allowed voltage step: 25 µV, maximum difference between any values: 100 µV, minimum/maximum amplitude: −100/100 µV), trials that showed horizontal eye movement (HEOG maximum difference between any values between −300 and 300 ms: 50 µV) were excluded. The average number of segments per subject that went into the final stages of the analysis was 164 ± 26 with a minimum of 94 segments. Independent component analysis was conducted in EEGLAB [16] removing any components, which were unequivocally related to eye movements, the light sensor or response pad artifacts. Finally, data was re-referenced to a global average as well as baseline corrected, before averaging across the LVF and RVF condition in each subject. N170 latencies were extracted from the most negative value between 100 ms and 300 ms in the PO7/PO8 electrode. Peak latency detection is commonly done in this or a similar fashion [43], [73], [12], [24], however, a clear peak as part of an ERP had to be identifiable. Selection of parietal electrodes is commonly used and has been recommended [7], [21]. Other studies have included PO7/PO8 in their analyses [2], [73], [24] and pilot testing yielded the most pronounced ERPs for PO7/PO8, which has also been reported before [1]. Subjects showing equivocal average waveforms were excluded from the analysis (see Table 1 for resulting N). IHTT was defined as the latency difference between the crossed and the uncrossed condition:

RVF: IHTT_left--->right_ = latency_PO8_ – latency_PO7_.
LVF: IHTT_right--->left_ = latency_PO7_ – latency_PO8_.

**Table 1:** Descriptive characteristics and distribution of relevant variables across groups *p <.05.

Group	Parameter	N	Mean ± STD	Distribution
younger	age	23	25.09 ± 2.59 y	Shapiro-Wilk = 0.959, p =.439
	IHTT_left -->right_	21	41.29 ± 17.00 ms	Shapiro-Wilk = 0.946, p =.280
	IHTT_right -->left_	21	39.52 ± 14.44 ms	Shapiro-Wilk = 0.963, p =.572
older	age	107	67.69 ± 5.18 y	Shapiro-Wilk = 0.958, p =.002*
	IHTT_left -->right_	87	52.24 ± 27.75 ms	Shapiro-Wilk = 0.976, p =.109
	IHTT_right -->left_	88	49.10 ± 26.59 ms	Shapiro-Wilk = 0.963, p =.013*
older female	age	43	68.74 ± 5.70 y	Shapiro-Wilk = 0.962, p =.159
	IHTT_left -->right_	34	58.50 ± 26.77 ms	Shapiro-Wilk = 0.972, p =.517
	IHTT_right -->left_	35	50.17 ± 27.63 ms	Shapiro-Wilk = 0.952, p =.133
older male	age	64	66.98 ± 4.71 y	Shapiro-Wilk = 0.960, p =.038*
	IHTT_left -->right_	53	48.23 ± 27.87 ms	Shapiro-Wilk = 0.961, p =.080
	IHTT_right -->left_	53	48.40 ± 26.13 ms	Shapiro-Wilk = 0.963, p =.095

### Statistical analyses

Statistical analyses were conducted using Python 3.8 (Python [53] and JASP [30]) and significance was accepted at p <.05. Outliers were defined as exceeding the limits of 1.5-times the interquartile range below the first or above the third quartile respectively and were excluded case wise. In order to evaluate the concept of IHTT, analysis of variance (ANOVA) was performed with ERP latency as dependent variable and age group, electrode site and visual field as fixed factors. This was done regardless of normal distribution of the sample, as ANOVA has been shown to be highly robust in this regard [60]. Post-hoc testing was performed applying Bonferroni correction. Parameters, which were passed into further analyses included age, IHTT_left-->right,_ IHTT_right-->left_, RTs and RA with a group factor differentiating between younger and older as well as male and female groups. In order to verify that IHTT reflects actual transfer and not elongated build up of the N1 component, correlation analyses between IHTT and direct N1-latency were conducted [7]. IHTT_left-->right_ and IHTT_right-->left_ were treated as unrelated dependent variables and, thus, separate analyses were conducted. Furthermore, the older group was divided into a male and female group, while the younger was not due to insufficient sample size. Each variable was tested for normal distribution across their respective group using the Shapiro-Wilk-Test. Subsequent group comparison analyses employed the Welch’s *t*-test, as has been suggested previously [15], [70]. Correlation analyses among the older group employed Pearson’s product-moment correlation if normal distribution could be assumed while spearman’s rank correlation was used otherwise. Fisher’s z-transformation was conducted to compare correlation coefficients between groups. Correction for multiple testing was not done in order to avoid Type 2 error [54]. Effect sizes estimated by Cohen’s D (d) or Eta squared (η^2^) were calculated where applicable.

## 3 Results

**Fig. 2:**
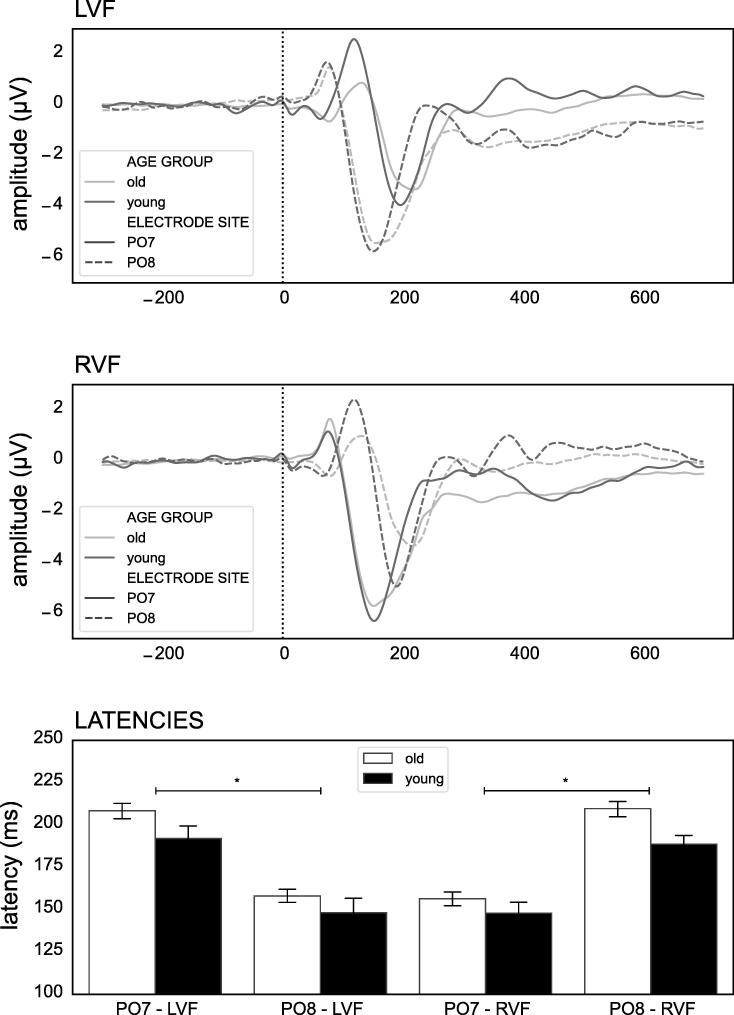
The grand averages of the relevant ERP components at PO7 and PO8 for the left visual field (A) and the right visual field (B) are depicted. Furthermore, the average latencies across the different conditions split by age group are illustrated (C). Significant differences are only indicated for the electrode site × visual field interaction effect for the relevant combinations, as age differences are analyzed using IHTT as dependent variable in separate analyses. *p <.05.

ANOVA revealed a significant group by electrode site by visual field interaction (ANOVA: F = 3.909, p =.049, η^2^ = 0.005) as well as a highly significant electrode site by visual field interaction (ANOVA: F = 384.450, p <.001, η^2^ = 0.447) and age group effect (ANOVA: F = 33.744, p <.001, η^2^ = 0.039). Post-hoc analyses showed elongated latencies for PO7 compared to PO8 when the stimulus was presented to the LVF (Bonferroni: t = 14.002, p <.001) and shorter ones when presented to the RVF (Bonferroni: t = -13.730, p <.001), respectively. Shorter latencies overall were found in the younger compared to the older group (Bonferroni: t = 5.809, p <.001, d = 0.433). Fig. 2 displays these results as well as the grand averages illustrating the concept of ERP-derived IHTT as found in the present sample.

Average IHTT_left-->right_ was found to be 50 ± 26 ms and IHTT_right-->left_ 47 ± 25 ms. Table 1 provides an overview of the extracted IHTT data split into the relevant groups divided by age and sex. Additionally, sample sizes within each group are shown after the exclusion of values connected to equivocal ERP components or outliers and the distribution for each parameter is displayed. Subsequent statistical testing was conducted accordingly.

Correlation analyses among all participants between IHTT and direct path N1 latency revealed significant negative correlations between IHTT_left-->right_ and latency_RVF-PO7_ (Spearman: r = −0.54, p <.001) as well as between IHTT_right-->left_ and latency_LVF-PO8_ (Spearman: r = −0.46, p <.001).

**Fig. 3:**
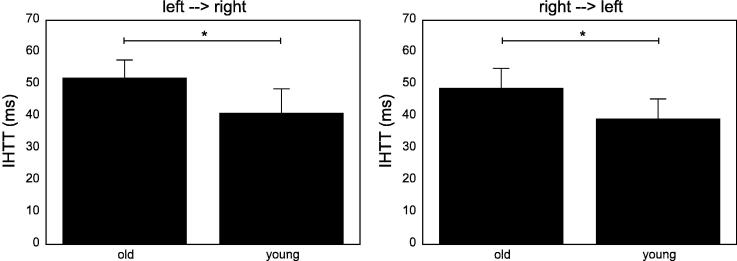
Averages of IHTT in both directions are shown for each age group. *p <.05.

Subsequent group comparisons revealed significantly shorter IHTT_left-->right_ (Welch: t = 2.304, p =.025, d = 0.476) as well as shorter IHTT_right-->left_ (Welch: t = 2.260, p =.028, d = 0.448) in the younger group as shown in Fig. 3.

**Fig. 4:**
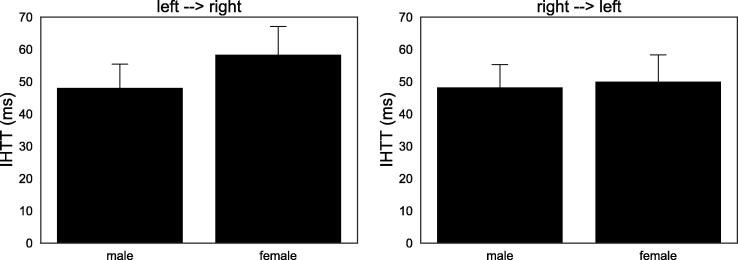
Averages of IHTT in both directions are shown for women and men in the older group. No significant differences were found.

Group comparisons between female and male participants among the older subjects revealed no significant difference, neither for IHTT_left-->right_ (Welch: t = 1.719, p =.090, d = 0.374) nor for IHTT_right-->left_ (Welch: t = 0.301, p =.764, d = 0.066). These results are illustrated in Fig. 4.

Correlation analyses within the older group revealed a significant correlation between age and IHTT_left-->right_ (Spearman: r = 0.22, p =.045), but a non-significant one between age and IHTT_right-->left_ (Spearman: r = 0.16, p =.143).

**Fig. 5:**
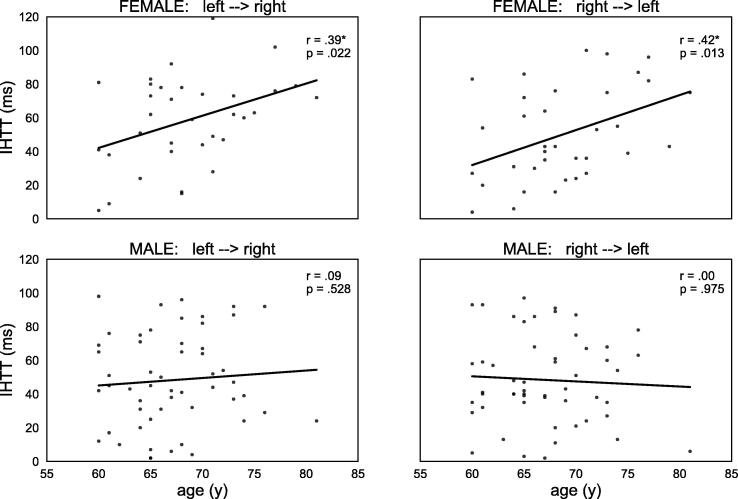
Scatterplots illustrating correlations between age and IHTT among female and male participants of the older group. *p <.05.

Correlation analyses within the female and male groups among the older subjects revealed a significant correlation between age and IHTT_left-->right_ (Pearson: r = 0.39, p =.022) as well as IHTT_right-->left_ (Pearson: r = 0.42, p =.013) in the female group. The same correlation could not be found for IHTT_left-->right_ (Spearman: r = 0.09, p =.528) or IHTT_right-->left_ (Spearman: r = 0.00, p =.975) in the male group. The correlation coefficients differed non-significantly between the two groups for IHTT_left-->right_ (Fisher: z = 1.407, p =.159), but significantly for IHTT_right-->left_ (Fisher: z = 1.995, p =.046). Illustrations are shown in Fig. 5.

## 4 Discussion

The main goal of the present study was to investigate the effect of aging on IHTT while considering potential sex differences. It is difficult to compare the extracted data to previous findings as studies have employed very different methods with regards to testing paradigms, ERP recording and analyses as well as statistical approach.

Analyses of latencies of the N170 components support the general concept of IHTT in the present study. This is indicated by a significant shorter latency of the N170 component in the hemisphere contralateral from the presented stimulus compared to the latency in the ipsilateral hemisphere. The size of this effect was large at η^2^ = 0.447 [39]. Thus, IHTT was analyzed further as separate variables for each direction. Correlation analyses between IHTT and direct path N1 latency reveal shorter N1 latencies corresponding to elongated IHTT. This indicates, that elongated IHTT is not caused by slower N1 build up in this sample, which has previously been identified as a prerequisite for the interpretation of IHTT as actually reflecting transfer [7]. A potential explanation for the negative relationship might be varying strategies with shorter N1 generation compensating for elongated interhemispheric transfer or vice versa. However, this idea of a compensatory mechanism needs to be investigated further using adequate methods in future research. The present study found rather large average IHTT values with an IHTT_left-->right_ of 50 ± 26 ms and IHTT_right-->left_ of 47 ± 25 ms, while based on previous reports it was estimated at 5–30 ms employing the ERP based approach along with the Poffenberger paradigm [40]. A systemic error seems unlikely, as the larger values were found for both directions in the expected conditions and only clearly identifiably ERPs were included into the analyses. This suggests that IHTT can be longer, than previously expected. This notion may be supported by similarly large values for IHTT in the younger group in a previous investigation [6] or comparable experiments in a different context [19], [2]. Furthermore, a rather large variability of IHTT was observed across both age groups, which has also been found in a previous investigation [6]. This variability may be natural, with more prominent differences in IHTT being mitigated by the different paths transfer can generally take [27]. It may also be increased by the impact of eye dominance. IHTT has been shown to be influenced by which eye is the dominant one interacting with handedness [10]. Right handers appear to show faster IHTT_right-->left_ only if their right eye was dominant and vice versa for dominance of the left eye. However, large variability should have no effect on our findings concerning age and sex effects, especially considering the present sample size.

### IHTT and age

Group comparisons in this sample revealed small effects showing significantly longer IHTT as well as generally elongated ERP latencies in older than younger subjects. Similarly, a significant, positive relationship between age and IHTT was found, though in this sample heavily driven by the female participants.

A number of previous studies employing the CUD method also found elongated IHTTs in older subjects [31], [55], [3], [62], although it should be noted that CUD and ERP derived measures of IHTT are not necessarily comparable [37]. The present finding of elongated IHTT in older subjects is also in line with one of the four other ERP studies, indicating age-related slowing of interhemispheric transfer [13], but not with the other three studies, which found no difference for N1-based IHTT for older compared to younger subjects [23], [6], [59].

However, the latter studies differed from the present one in a number of ways and may not be entirely suitable to provide evidence for or against potential effects of age in combination with gender. In the study by Boyson [6], the P1 component yielded even shorter IHTT in older subjects than in the younger ones, though the opposite was expected from the available literature. The author further mentioned rather noisy data and further investigation and replication was recommended [6].

The study by Hoptman et al. [23] relied on very low-density ERP recordings (i.e., only 6 electrodes), while also including more temporal electrode sites in the estimation of IHTT [23]. In contrast, the present study used 64 electrodes for artifact rejection algorithms, independent component analysis and global referencing as well as generally more advanced hardware and the more conventional parietal electrodes. Additionally, the use of temporal electrode sites in the study by Hoptman et al. [23] may obscure age-related elongation of IHTT. This is because IHTT estimations have been shown to be shorter in anterior and temporal regions [29].

Scally et al. [59] employed ERP detection in young (18–27) and older adults (63–80) constrained to the alpha and theta frequency band [59]. In this case an age-effect was not inferred due to the effect being driven by decreased contralateral as opposed to increased ipsilateral N1 latency. Although benefits from shorter transfer with completion at the same time due to increased contralateral latency in the younger participants are questionable, as was pointed out [59], shorter IHTT in this context should not be considered unimportant. Future studies may want to focus on potential compensatory effects [65]. For example, slower IHTT could be compensated for by earlier sensory processing in older adults [59], however it remains to be seen if this mechanism is sustainable or only applicable to early stages of aging. The present results of a negative relationship between IHTT and direct path N1 latency are indicative of some such mechanism. At any rate, elongated IHTT should not be disregarded as a factor related to aging, even if overall latencies remain unaffected. Additionally, increased latency in younger participants as seen in the study of Scally et al. [59], appears counter intuitive and was not confirmed in studies using traditional ERP approaches [23], [13]. As assumed by Scally et al. [59], the shorter latencies in older subjects may be limited to their constrained ERP approach. Increased latencies in older participants such as the small but significant effect found in the present investigation are to be expected [13], [14]. Finally, no age-related effect was expected by Scally et al. [59], due to the general assumption, that age-effects of the CC’s structure are more pronounced in anterior than posterior regions, with the latter being responsible for transfer of visual information. However, although some degree of preservation of posterior regions of the CC in normal aging has been found, by no means are those regions completely spared, such that subtle but increasing age-related degradation of posterior parts of the CC has been suggested [49]. Structural decline of the CC has been shown in posterior regions [46] and despite the tendency towards faster decline in anterior parts, IHTT may still be affected by these or any other means yet unknown.

Thus, considering the few studies addressing IHTT in the context of aging and considering the respective methodological differences with previous studies, the present results support the notion of an age-related elongation of IHTT.

### Impact of sex

The effects of sex in the context of aging have to be assessed differentially. Group comparisons in the present investigation showed no difference between men and women, while correlation analyses indicated a positive relationship between age and IHTT, driven by the female group. This is in line with previous investigations, although the basis for this notion is complex.

One study found age-related decline using the CUD method and described the aging effect being largely driven by the female participants [31]. Although direct comparisons of IHTT between men and women failed to reach significance in the present study, a similar trend was apparent, especially when considering the positive correlation between IHTT and age in women. Interestingly, other studies investigating much younger participants actually found shorter IHTT estimates in women than men [44], [43]. All of these findings are not necessarily contradictory. A possible explanation may be that the difference in IHTT between women and men depends on the average age of the sample. Considering the previous findings in this context, this may be due to women displaying shorter IHTT at younger ages, but earlier or faster detectable age-related decline compared to men. Increased elongation of IHTT at certain age ranges has been demonstrated before [3], although in this case, sex differences were found for auditory transfer times, but not for visuo-motor transfer times, measured using the CUD method. A similar effect may be possible for ERP derived IHTT in the visuo-motor domain.

Although, the present findings found significantly different relationships between IHTT in women and men only for one direction (IHTT_right-->left_), the overall relationship between IHTT and age appears to be driven by the female participants in this sample. The present results are supported by findings of structural degradation of the CC, which may be more prominent in older women [58], [71], [36]. A relationship between IHTT and white matter integrity of the CC is plausible and has been indicated by previous findings [73], [24]. Thus, sex-related differences in structural integrity of the CC may be one factor causing similar differences with regards to IHTT.

Future research should also focus on the impact of postmenopausal, hormonal alterations in this context. Female hormones may have beneficial effects with regards to interhemispheric communication [67] and, consequently, menopause may be a relevant factor in the context of the sex-related differences in the present results.

In summary, the present findings are indicative of a stronger ongoing elongation of IHTT in women at the age range of the present sample (i.e., 60-81y), which could be related to differences in CC’s structure as well as its interaction with aging. Post-menopausal alterations could be a relevant factor and require further investigation. This concurs with some previous findings, although further investigation with the concrete goal of examining sex-related differences of IHTT in the context of aging is necessary.

### Limitations

The present study is subject to some limitation. In order to receive a certain degree of comparability, an established letter matching task was chosen. However, this does not allow for the analysis of CUD derived IHTT and complicates inference and evaluation in the light of previous research. The comparison of IHTT based on ERPs versus CUD is questionable [59] and a more solid basis of ERP derived estimates of IHTT in the context of aging is vital for reliable interpretation.

Furthermore, we used PO7/PO8 electrodes to increase comparability to a number of studies, although this approach may not fully represent anatomical areas. However, we expect close enough approximation and prior pilot testing yielded most prominent ERPs using this approach.

Additionally we failed to control for eye dominance as has been suggested previously [10]. In most cases this is especially relevant, as eye dominance is unevenly distributed among right-handers [4]. However, as we only investigated age and sex effects as opposed to asymmetry, we do not expect eye dominance to have an impact on our findings.

Common peak latencies were used to estimate IHTT, while potentially more accurate methods have been suggested [35]. However, at the same time the authors acknowledge that the optimal approach depends on the unambiguousness of the waveform and the present study only included clearly identifiable peaks. The conservative approach with regards to ERP identification in combination with the relatively large sample size should mitigate the drawbacks related to individual peak-based latency estimation.

Only cross-sectional analyses with a narrow range with regards to age were conducted, due to limited resources. Previous research and the present study have shown, that the impact of aging on IHTT may be complex and interact with other factors such as sex while being more prominent at certain stages along the aging process than others [3]. Including subjects with a broader range of ages or even longitudinal data may have been able to better pinpoint relevant elongation of IHTT.

Finally, the younger group was much smaller, than the older, again, due to limited resources. The main goal of this study was to explore IHTT in aging and a relatively larger group of older subjects helped to increase power in the correlation analyses. However, more young participants may have been able to more clearly discern the effects of sex in the aging context. Allowing for the estimation of the relationship between IHTT and sex in the younger group may have enabled the investigation of potential differences of said relationship in the older group. To date, this relationship between IHTT and sex itself is not sufficiently understood [43], such that inference with regards to sex modulating aging effects remains speculative.

## Conclusion and future directions

The present results support the idea of IHTT as a useful tool to estimate interhemispheric processing across the CC [43], [45], [73], as it provides a rather unequivocal estimate of the CC’s functionality. The present findings suggest, that IHTT is subject to age-related elongation. Furthermore, the decline of transfer speed occurs differently between men and women, with women experiencing a stronger decline at roughly the age of the present older group (60-81y). Future investigations should include a broader range of ages among subjects or even longitudinal assessment in order to better pinpoint any occurring increase of IHTT. Additionally, other factors known to influence aging effects on the CC, such as physical activity [32] should be accounted for. Finally, a better understanding of the relationship between IHTT and structural properties of the CC is required, such that the significance of IHTT as a simple in vivo estimate of CC integrity becomes more assessable.

## Conflict of interest statement

The authors declare that the research was conducted in the absence of any commercial or financial relationships that could be construed as a potential conflict of interest.
